# The economic burden of pulmonary arterial hypertension (PAH) in the US on payers and patients

**DOI:** 10.1186/s12913-014-0676-0

**Published:** 2014-12-24

**Authors:** Mirko Sikirica, Serban R Iorga, Tim Bancroft, Jesse Potash

**Affiliations:** Value Evidence and Outcomes, GlaxoSmithKline, 2301 Renaissance Blvd, King of Prussia, PA, 19406 USA; Health Economics and Outcomes Research, Optum, 12125 Technology Drive, Eden Prairie, MN 55344 USA; Market Access and Value Strategy, Optum, 12125 Technology Drive, Eden Prairie, MN 55344 USA

**Keywords:** PAH, Costs, Resource use, Retrospective, Managed care, Pulmonary arterial hypertension

## Abstract

**Background:**

Pulmonary arterial hypertension (PAH) is a rare condition that can ultimately lead to right heart failure and death. In this study we estimated the health care costs and resource utilization associated with PAH in a large US managed care health plan.

**Methods:**

Subjects with claims-based evidence of PAH from 1/1/2004 to 6/30/2010 (identification period) were selected. To be included in the final PAH study sample, subjects were required to have ≥2 claims with a primary PH diagnosis; ≥2 claims with a PAH related-diagnosis (connective tissue diseases, congenital heart diseases, portal hypertension); and ≥1 claim with evidence of a PAH-indicated medication. The earliest date of a claim with evidence of PAH-indicated medication during the identification period was set as the index date. Health care costs and resource utilization were compared between an annualized baseline period and a 12 month follow-up period.

**Results:**

504 PAH subjects were selected for the final study cohort. Estimated average total health care costs were approximately 16% lower in the follow-up period compared to the baseline period (follow-up costs = $98,243 [SD = 110,615] vs. baseline costs = $116,681 [SD = 368,094], p < 0.001), but substantively high in each period relative to costs reported for other chronic diseases. Pharmacy costs were significantly higher in the follow-up period vs. the baseline period, ($38,514 [SD = 34,817] vs. $6,440 [SD = 12,186], p < 0.001) but medical costs were significantly lower in the follow-up vs. baseline ($59,729 [SD = 106,683] vs. $110,241 [SD = 368,725], p < 0.001). These costs were mirrored in health-care resource utilization estimates. The average counts of ambulatory visits and inpatient stays were lower in the follow-up vs. the baseline (both p < 0.001). Results varied in exploratory analyses when less restrictive subject identification algorithms were used.

**Conclusions:**

Subjects with evidence of PAH had substantively high health care costs. Medical costs appeared to decrease following PAH medication use, but with a concomitant increase in pharmacy costs.

**Electronic supplementary material:**

The online version of this article (doi:10.1186/s12913-014-0676-0) contains supplementary material, which is available to authorized users.

## Background

Pulmonary arterial hypertension (PAH) is one of five groups of pulmonary hypertension (PH) classified by the 2009 European Society of Cardiology and European Respiratory Society guidelines [1]. PAH is characterized by constriction of pulmonary arteries and an increase in pulmonary vascular resistance, leading to right heart failure [2]. Abnormal proliferation of smooth muscle and endothelial cells is thought to play an important role in disease pathology [3]. The hemodynamic profile of PAH is described as a mean pulmonary arterial pressure of 25 mmHg or higher, and a pulmonary capillary wedge pressure of 15 mmHg or lower [4]. Within the PAH category, several subgroups of disease are recognized, including idiopathic PAH, heritable PAH (links to genetic mutations in BMPR2 [5] and ALK-1 [6] have been found), connective tissue disease-associated PAH (e.g., PAH associated with systemic sclerosis or systemic lupus erythematous), PAH associated with congenital heart disease, and portopulmonary hypertension [7]. Diagnosing the underlying causes of various types of PH and PAH is challenging, and is facilitated by classification guidelines [8]. Diagnosis of PAH has been found to be more common among women than men [9], and survival among PAH patients has been reported at 83% at 1 year and 58% at 3 years [10].

Symptoms of PAH include dyspnea on exertion, fatigue, chest pain, and fainting [2]. Although there is no cure for PAH, pharmacotherapy may be used to manage the disease and improve symptoms [4]. Several classes of pharmacotherapy are available to treat PAH: endothelin receptor antagonists (bosentan and ambrisentan); phosphodiesterase type 5 inhibitors (sildenafil and tadalafil); and prostacyclin analogues (epoprostenol, treprostinil and iloprost) [11]. Studies have demonstrated that combination therapy with 2 or more medications from different classes may provide benefit over monotherapy [12,13]. As symptoms of PAH are similar to those of other diseases, diagnosis may be delayed until more advanced disease stage, when treatment is not as successful [14]. Surgical intervention, such as lung transplant or balloon atrial septostomy, may be needed for patients who are not adequately controlled with medication [4].

PAH is a rare but costly disease. The prevalence of PAH in the US has been estimated at 109 per million among individuals under 65, and 451 per million among individuals 65 and over [15]. Limited information is available regarding real-world health care resource utilization patterns and costs among PAH patients in the US, in part due to the difficultly in identifying PAH using administrative claims data. Copher et al. reported average all-cause health care costs among patients with PAH at $9,295 per-patient-per-month (PPPM) [16], Angalakuditi reported average PAH-related health care costs at $4,236 PPPM [17], Said et al. reported average total health care costs among patients with PAH at $4,021 PPPM [18], and Kirson et al. reported average PPPM direct health care costs for PAH at $2,023 [19]. As existing ICD-9-CM codes may not be specific enough to distinguish PAH from other related conditions, prior studies have used a variety of approaches and different combination of claims-based evidence to identify PAH patients from administrative databases, including diagnosis claims for PH, evidence of a right heart catheterization, and claims for medications used to treat PAH.

The objective of this study was to explore the health care costs and resource utilization associated with PAH in a large, national US managed care health plan using contemporary real-world data with a substantial time frame (five and one-half years). In this study, we required that PAH subjects have claims-based evidence of PAH-indicated medications, PH diagnoses, and PAH-related diagnoses in order to make subject selection as specific as possible. By setting the index date as the earliest date of evidence for a PAH-indicated medication (and then performing comparisons between the baseline and follow-up periods), we hoped to gain additional insight into how PAH medication use may influence total health care costs among PAH patients. Additionally, in this study we examined both health plan and patient paid costs, to better understand the economic burden on payers and patients, respectively.

## Methods

### Data source and subject selection

This study used administrative claims of commercial and Medicare Advantage enrollees with Part D prescription drug coverage from a large national managed care organization. During the period of the study, the database contained approximately 41.7 million members with continuous medical and pharmacy benefits (and approximately 14 million total covered lives per year). The geographic makeup of individuals covered by the health plan was diverse, with services rendered in all major regions of the United States. No identifiable protected health information was extracted or accessed during the course of the study. Pursuant to the Health Insurance Portability and Accountability Act [20], the use of de-identified data does not require Institutional Review Board approval or waiver of authorization, and research involving the collection or study of already existing de-identified data does not require patient consent [21].

**Figure 1:**
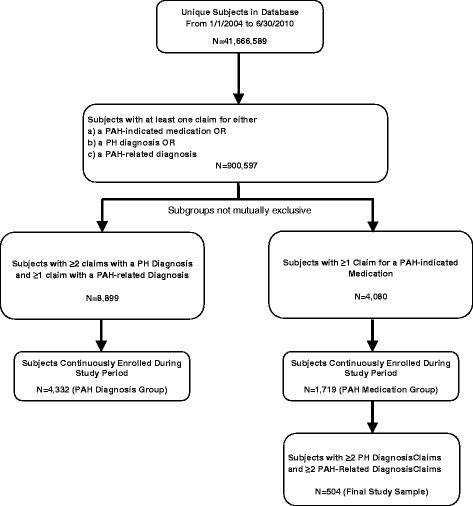
**Selection of subjects with PAH.**

Subjects were initially identified from the database who had at least 1 claim during the period from 01 January 2004 to 30 June 2010 (defined as the identification period) for either a PAH-indicated study medication (bosentan, ambrisentan, tadalafil [Adcirca®], sildenafil [Revatio®], iloprost, treprostinil, epoprostenol); an ICD-9-CM diagnosis code in any position for primary pulmonary hypertension (416.0) or other chronic pulmonary heart diseases (416.8); or a diagnosis code in any position for a PAH-associated condition (connective tissue diseases, congenital heart diseases, portal hypertension; codes in Additional file 1: Table S1). After this initial selection step, subjects who had ≥2 claims with a primary PH diagnosis, ≥2 claims with a PAH-related diagnosis (of which ≥1 primary), and ≥1 claim with evidence for a PAH-indicated medication were selected for the final study sample (Figure 1). The index date for the final study sample was designated as the earliest date of a medical or pharmacy claim with evidence of PAH-indicated medication in the identification period. For inclusion in the final study sample subjects were required to be continuously enrolled during a 180-day baseline period before the index date, and for an additional 365-day follow-up period starting on and including the index date.

Exploratory analyses were also performed, in which subjects were also eligible for selection into two subgroups with less stringent inclusion criteria: a PAH diagnosis group and a PAH medication group (Figure 1). Overlap of subjects between the PAH diagnosis group and the PAH medication group was allowed. Criteria required for inclusion in the PAH diagnosis group were evidence of ≥2 medical claims with a PH diagnosis code at least 15 days apart (of which ≥1 was in the primary position) and ≥1 medical claim with a (primary or secondary) PAH-related diagnosis during the identification period. The index date for the PAH diagnosis group was designated as the earliest date of a medical claim with a diagnosis code in any position for either PH or a PAH-related condition during the identification period. Inclusion in the PAH medication group required ≥1 instance of evidence for a PAH-indicated medication. The index date for the PAH medication group was designated as the earliest date of a medical or pharmacy claim with evidence of PAH-indicated medication in the identification period. Subjects in both the PAH diagnosis group and the PAH medication group were required to be continuously enrolled during a 180-day baseline period before the index date, and for an additional 365-day follow-up period starting on and including the index date.

Age, gender, and geographic region (Northeast, South, West, or Midwest) were captured from enrollment data based on the claim used to identify the index date. Comorbidities were identified based on the presence of diagnosis and/or service codes on medical claims during the baseline and follow-up periods, using AHRQ classifications [22]. Each AHRQ category was comprised of a series of diagnosis codes corresponding to similar disease conditions. Presence of any code within a given category in any position during a given time period would set the indicator variable to 1. In contrast, absence of all codes within a category across all claims during a given time period would set the indicator variable to 0.

### Health care costs and resource utilization

Health care resource utilization and costs were captured in the baseline and follow-up periods. The baseline continuous measures were annualized, to provide comparable metrics to the follow-up period. Health care costs were computed as the combined health plan and patient paid amounts; in addition, payments from Medicare (or other payers) were estimated based on coordination of benefits information obtained by the health plan, and these estimates were incorporated into the total paid amount. The following cost variables were calculated: total costs, pharmacy costs, and medical costs. Medical costs were further broken down into ambulatory costs, emergency services costs, inpatient costs, and other medical costs. Costs were presented as total (health plan plus patient paid), health plan paid and patient paid. Patient paid costs were comprised of the fixed amount that a member paid for a specific service as defined in their benefit plan, the amount that was applied towards the member’s deductible, and any additional dollars paid by a member at the pharmacy (above copay, coinsurance or deductible). Procedures, drug dispensings, and doctor visits that occurred during an inpatient admission were included in the costs for that admission (the same applied to costs incurred during an emergency department or ambulatory visit). Costs were adjusted to 2011 US$ using the annual medical care component of the Consumer Price Index. Health care resource utilization was operationalized as the numbers of the following: ambulatory visits, emergency department visits, and inpatient admissions. Comparisons of continuous variables were made using signed rank test. All software analyses were conducted in SAS 9.2.

### Medication use and treatment patterns

Medications that could be used to treat PAH were identified from pharmacy claims (using National Drug Code [NDC] code bundles) and medical claims (using appropriate Healthcare Common Procedure Coding System [HCPCS] codes submitted by individual providers which use the Health Care Financing Administration [HCFA]-1500 or Centers for Medicare and Medicaid Services [CMS]-1500 formats or for facility services submitted by institutions, which use the Uniform Bill [UB]-82, UB-92, UB-04, or CMS-1450 formats). Medication use was captured separately in both the baseline and follow-up periods.

Medication treatment patterns were captured across follow-up period pharmacy claims, and only for those subjects who had evidence of a single PAH-indicated study medication in the first 30 days of the follow-up period. Discontinuation of index therapy was defined as the earliest gap (if multiple exist) in therapy of >60 days between the “run out” of days supplied from the previous prescription (i.e., prescription fill date + days supplied – 1) to the fill date of the next prescription or the end of follow-up. If a subject had a 60-day gap in index therapy, the discontinuation date was noted as the run out date. Subjects whose last run out date in a series of continuous use was within 60 days of the end of the study period were considered continuous users of therapy with no evidence of discontinuation. Switches from index PAH treatment at the time of discontinuation were assessed. A switch in therapy was defined as a filled prescription for a new PAH medication that occurred within 60 days prior to or following the date of index PAH medication discontinuation. PAH treatment add-ons were identified for patients adding new PAH therapies to their index therapy while continuing use of index therapy. Add-on was defined as the addition of a new PAH medication other than the index medication at least 60 days prior to the date of discontinuation or 60 days prior to the end of the study period if there was no evidence of discontinuation. Subjects who did not discontinue, switch, or add-on to their index therapy were classified as having maintenance of index therapy. Finally, the medication possession ratio (MPR) was captured and reported separately for each PAH index therapy. MPR was defined as the proportion of follow-up days with the index therapy available based on fill dates and day supply given on the pharmacy claims. MPR was calculated as the number of non-inpatient days where the medication of interest was available (numerator) divided by the total number of non-inpatient days (denominator). MPR was captured until discontinuation for subjects who discontinued; for those who didn’t discontinue, MPR was captured during the entire 12 month follow-up period.

## Results

In total, there were 41,666,589 enrollees in the health plan during the study period. Of these, 504 PAH subjects who met continuous enrollment criteria and had claims-based evidence of PAH-indicated medications, PH diagnoses, and PAH-related diagnoses were selected for the final study sample (Figure 1). In the final PAH study sample, subjects had an average age of 49.73 years, and 23.21% of subjects were male. Approximately three-quarters of subjects were from either the South or Midwest (which reflects the geographic distribution of health plan enrollees), and 87.10% of subjects had commercial insurance (Table 1). The most frequently observed comorbidities, based on claims data, were diseases of the heart (97.42% of subjects), lower respiratory disease (85.91%), hypertension (59.13%), diseases of arteries, arterioles, and capillaries (52.98%), and systemic lupus erythematosus and connective tissue disorders (51.79%) (Table 1). We note that some comorbidities identified here, such as diseases of the heart and lower respiratory diseases, are symptoms of PAH. It is likely that the comorbidity profile is partly a reflection of the PAH-associated conditions included in the subject identification algorithm.

**Table 1:** **Subject demographic and clinical characteristics**

	**Final PAH study sample (N = 504)**	
	**Mean**	**SD**
Age, years	49.73	19.79
	**n**	**%**
Male gender	117	23.21
Geographic region		
Northeast	45	9.15
Midwest	148	30.08
South	216	43.90
West	83	16.87
Insurance		
Commercial	439	87.10
Medicare	65	12.90
Baseline comorbidities		
Diseases of the heart	491	97.42
Lower respiratory disease	433	85.91
Hypertension	298	59.13
Diseases of arteries, arterioles, and capillaries	267	52.98
Systemic lupus erythematosus and connective tissue disorders	261	51.79
Respiratory infections	188	37.30
Upper gastrointestinal disorders	182	36.11
Non-traumatic joint disorders	181	35.91
Connective tissue disease	170	33.73
Injuries and conditions due to external causes	163	32.34
Diseases of the urinary system	162	32.14
Chronic obstructive pulmonary disease and bronchiectasis	156	30.95
Disorders of lipid metabolism	153	30.36
Gastrointestinal disorders	141	27.98
Cardiac and circulatory congenital anomalies	136	26.98
Anemia	133	26.39

**Figure 2:**
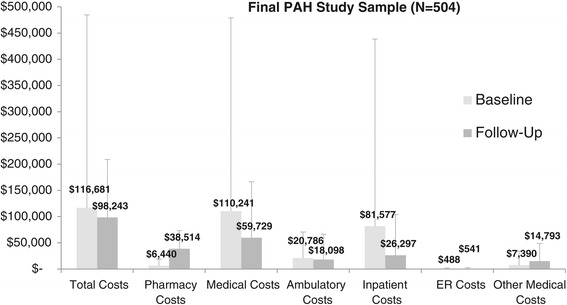
**Average health care costs (annualized baseline and 12 month follow-up).** Differences in costs between baseline and follow-up were statistically significant (p < 0.001) for all categories, with the exception of ER costs (p = 0.700). Bars represent standard deviation.

**Figure 3:**
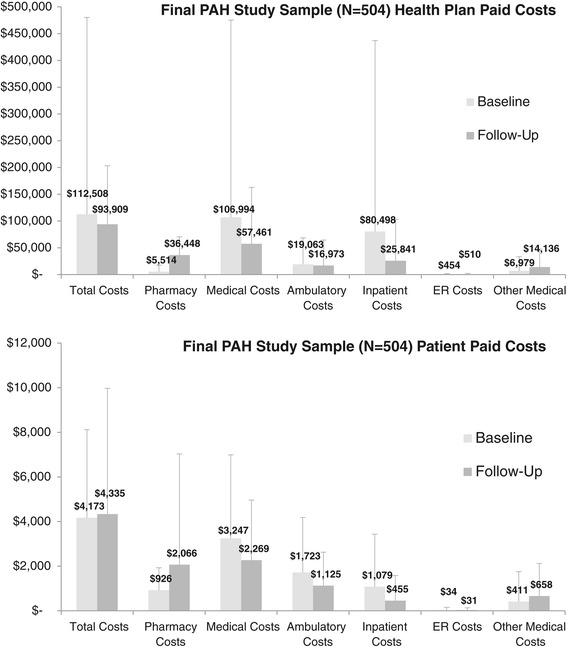
**Average health plan and patient paid health care costs (annualized baseline and 12 month follow-up).** For health plan paid costs, differences in costs between baseline and follow-up were statistically significant (p < 0.001) for all categories, with the exception of ER costs (p = 0.653). For patient paid costs, differences in costs between baseline and follow-up were statistically significant (p < 0.001) for all categories, with the exception of total costs (p = 0.989) and ER costs (p = 0.758). Bars represent standard deviation.

Among patients in the final study cohort (N = 504), the average of the total annual health care costs were higher in the baseline period ($116,681 [SD = 368,094]) vs. the follow-up period ($98,243 [SD = 110,615]) (p < 0.001) (Figure 2). Whereas the average medical costs were significantly higher in the baseline period vs. the follow-up period ($110,241 [SD = 368,725] vs. $59,729 [SD = 106,683], p = <0.001), the average pharmacy costs were significantly lower in the baseline period vs. the follow-up period ($6,440 [SD = 12,187] vs. $38,514 [SD = 34,817], p < 0.001). Among medical costs, the averages of the inpatient costs were significantly higher in the baseline period vs. the follow-up period ($81,577 [SD = 357,009] vs. $26,297 [SD = 77,462], p < 0.001). The average ER costs were similar between the baseline period vs. the follow-up period ($488 [SD = 1,252] vs. $541 [SD = 1,774], p = 0.700), and average ambulatory costs were slightly higher in the baseline period vs. the follow-up period ($20,786 [SD = 49,781] vs. $18,098 [SD = 48,152], p < 0.001). In general, similar trends were observed when limiting analysis to health plan paid or patient paid costs. For both patient paid and health plan paid costs, medical costs were significantly higher in the baseline period vs. the follow-up period, whereas pharmacy costs were significantly lower in the baseline period vs. the follow-up period (Figure 3).

**Figure 4:**
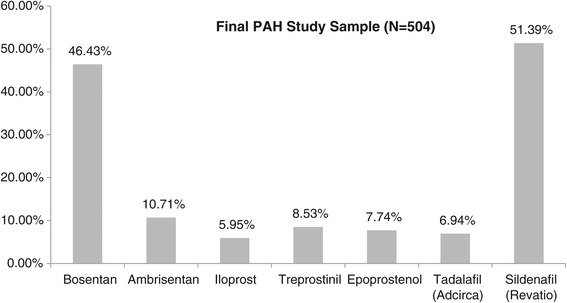
**PAH medication use (12 month follow-up).**

Patterns of health care resource utilization between the baseline period and follow-up period were consistent with the patterns observed for health care costs. Average annual counts of inpatient stays (1.38 [SD = 2.03] vs. 0.91 [SD = 1.59], p < 0.001), ambulatory visits (44.95 [SD = 26.86] vs. 41.62 [28.71], p < 0.001), and ER visits (1.56 [SD = 2.95] vs. 1.55 [SD = 3.17], p = 0.032) were higher in the baseline period vs. the follow-up period (Table 2). During the follow-up period, the most commonly used PAH-indicated medications were sildenafil (Revatio), used by 51% of subjects, and bosentan, used by 46% of subjects (Figure 4). About 31% of subjects had evidence of using more than one PAH-indicated medication during the follow-up period (data not shown). Treatment patterns were examined in greater detail using pharmacy claims for those subjects who had claims for only a single PAH medication during the first 30 days of follow-up (N = 425). Within this group, maintenance of the index therapy regimen was observed for 52.71% of subjects, and discontinuation was observed for 38.12% of subjects (Table 3). Of subjects who discontinued, 14.20% had evidence of switching from the index medication. Also, 11.06% of subjects had evidence of adding to the index medication. Average MPRs were over 0.90 for each individual PAH medication measured (standard deviations all < 0.13), except for iloprost (0.86 [SD = 0.21]), indicating good overall adherence to medication.

**Table 2:** **Health care resource utilization (annualized baseline and 12 month follow-up)**

	**Final PAH study sample (N = 504)**						
	**Baseline**			**Follow-up**			**p-value**
	**Mean**	**SD**	**Median**	**Mean**	**SD**	**Median**	
Health care utilization counts							
Ambulatory visits	44.95	26.86	42.58	41.62	28.71	36.00	<0.001
Office visits	26.17	18.74	22.31	24.63	18.01	21.00	0.010
Outpatient visits	19.19	16.56	16.22	17.34	18.49	12.00	<0.001
ER visits	1.56	2.95	0.00	1.55	3.17	0.00	0.032
Inpatient stays	1.38	2.03	0.00	0.91	1.59	0.00	<0.001

**Table 3:** **PAH medication treatment patterns (12 month follow-up)**

	**PAH subjects** ^**1**^ **(N = 425)**	
	**n**	**%**
Discontinuation of index therapy regimen	162	38.12
Switch from index therapy regimen^2^	23	14.20
Augmentation to index therapy regimen	47	11.06
Maintenance of index therapy regimen	224	52.71
Medication Possession Ratio (MPR)	**n**	**mean**
Bosentan	177	0.94
Ambrisentan	29	0.94
Iloprost	4	0.86
Treprostinil	2	1.00
Epoprostenol	0	-
Tadalafil (Adcirca®)	22	0.94
Sildenafil (Revatio®)	191	0.92

^1^Of the subjects in the final sample, 425 were monotherapy pharmacy users in the 30 days following the index date; these subjects were included in the analysis of follow-up treatment patterns.

^2^Percentage of switchers calculated out of subjects who discontinued (n = 162).

**Figure 5:**
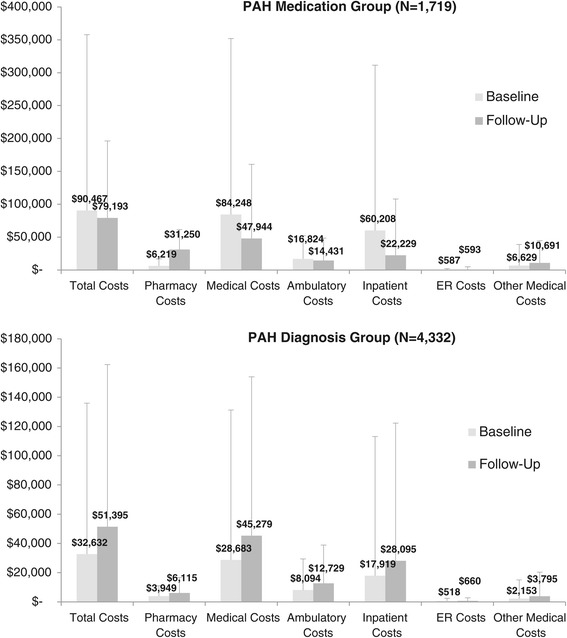
**PAH medication group and PAH diagnosis group average annual health care costs (exploratory analysis).** For PAH medication group, differences in costs between baseline and follow-up were statistically significant (p < 0.01) for all categories. For PAH diagnosis group, differences in costs between baseline and follow-up were statistically significant (p < 0.001) for all categories. Bars represent standard deviation.

In exploratory analyses that had less restrictive inclusion criteria, 4,332 subjects were selected who met continuous enrollment criteria and had claims-based evidence of PH diagnoses and a PAH-related diagnosis (PAH diagnosis group), and 1,719 subjects were selected who met continuous enrollment criteria and had claims-based evidence of a PAH-indicated medication (PAH medication group) (Figure 1). Average total baseline and follow-up health care costs among both the PAH medication group and the PAH diagnosis group were substantively lower than was observed among subjects in the final study cohort (Figure 5). Trends observed in the PAH medication group exploratory analysis were similar to those for the final study sample. In the PAH medication group analysis, medical costs were significantly higher in the baseline vs. the follow-up period (p < 0.001), while pharmacy costs were significantly higher in the follow-up vs. baseline period (p < 0.001) (Figure 5). However, in the PAH diagnosis group exploratory analysis (where the index date was set based on a diagnosis claim, not a PAH medication claim), total costs, pharmacy costs, and medical costs were all higher in the follow-up period vs. the baseline period (all p < 0.001) (Figure 5).

## Discussion

In this study we investigated the health care costs and resource use associated with PAH using a large, national US managed care health plan database. The index date in this study was set as the earliest date of evidence for a PAH-indicated medication, and health care costs were compared before and after the index date. Cost estimates for PAH in the final study sample were substantively high, and were calculated to be $116,681 in the baseline period and $98,243 in the follow-up period. We found that although pharmacy costs increased significantly from the baseline to follow-up period, medical costs decreased significantly from the baseline to follow-up period. The decrease in medical costs appeared to be driven primarily by inpatient costs, which decreased by more than two-thirds from baseline to follow-up. Importantly, the index date for the final PAH study cohort was set as the earliest date of a claim with evidence of PAH-indicated medication during the identification period. A possible interpretation of these trends is that although medication costs for PAH subjects are high, pharmaceutical management with a PAH-indicated medication is associated with a reduction in medical costs among subjects with disease. However, due to the observational study design, it was not possible to establish a direct causal relationship between PAH medication use and pre-index/post index changes, such as a reduction in medical costs.

The differences observed for health care costs were reflected in health-care resource utilization estimates, as average counts of ambulatory visits, inpatient stays, and ER visits were lower in the follow-up period vs. the baseline period. Sildenafil (Revatio®) and bosentan were the most commonly prescribed PAH medications during the follow-up period among subjects in the final PAH study sample. Although evidence of PAH-indicated medication use appeared to offer benefit in this sample (based on health care cost and resource use trends from baseline to follow-up), only about half of PAH subjects maintained their index medication regimen. Discontinuation of the index regimen was observed for about 38% of subjects, and about 11% of subjects had evidence of augmentation. Future research could investigate PAH costs and treatment patterns among specific subgroups of patients, such as a patient population stratified by age or a population stratified by gender.

Several prior studies have investigated the economic burden of PAH in the US. The total health care costs among PAH subjects reported in this study are higher than those reported by Said et al. In a retrospective study using administrative claims data, Said et al. found that the total health care costs of PAH patients identified from 2004–2009 were $4,021 per-patient-per-month (PPPM) [18]. By comparison, we found that average total health care costs were about $98,243 for the 12 month follow-up period (or about $8,187 per month). Unlike this study, Said et al. required evidence of right heart catheterization or echocardiogram for subject inclusion. Importantly though, Said et al. did not require evidence of a PAH-indicated medication for subject inclusion, whereas we required that subjects have at least one claim for a PAH-related medication for inclusion in the final sample. This may in part explain the difference in costs between studies, as further analyses performed here indicated that the PAH-indicated medication requirement appeared to be more specific than the PH/PAH-associated diagnosis code requirement in selecting PAH subjects. Similarly, Kirson et al. reported that average PPPM costs among PAH patients identified from 2002–2007 were $2,023, and they also did not require evidence of PAH-indicated medication for subject inclusion [19]. The costs reported by Said et al. and Kirson et al. are closer to the costs reported here in the exploratory analysis for the PAH diagnosis group ($51,395 for the 12 month follow-up, or about $4,283 per month).

However, the costs reported in the present study are similar to costs for PAH determined by Copher et al. In a retrospective claims-based analysis, Copher et al. reported that the total health care costs of PAH patients identified from 2004–2008 were $9,295 per-patient-per-month [16], which is slightly higher than our estimate of about $8,187 per month. Similar to the present study, Copher et al. required both a PH diagnosis and evidence of PAH medication use for study inclusion. However, they also required evidence of a right heart catheterization or pulmonary hypertension-related inpatient stay for study inclusion, whereas PAH-related diagnosis claims were required in the present study. Similar to the present study, Copher et al. found that sildenafil and bosentan were the most common index medications. However, Copher et al. did not compare medical costs before and after an index medication date. In the present study, we found that medical costs were significantly lower in the follow-up period, compared with the baseline period, suggesting that PAH medication use may affect the costs associated with treating PAH. Angalakuditi et al. also included evidence of PAH-indicated medication in their subject identification algorithm (patients were identified from 2006–2008). However, Angalakuditi et al. reported on PAH-related healthcare costs ($4,236 PPPM) [17], and not all-cause costs as reported in the present study, so direct comparison between studies is not possible. Also, cost estimates in Angalakuditi et al. were limited to subjects with bosentan or sildenafil as the index medication. While bosentan and sildenafil appeared to be the two most commonly prescribed medications in the present study, PAH subjects who had evidence of using other medications (ambrisentan, tadalafil, iloprost, treprostinil, epoprostenol) were also included in the present study. In another recent study using a patient identification period from 2005–2008, Berger et al. compared medical and pharmacy costs of PAH patients before and after initiating sildenafil [23]. In comparisons between 6 month pretreatment and follow-up periods, Berger et al. reported an increase in pharmacy costs ($9,853 vs. $16,971), and a decrease in medical costs ($30,104 vs. $27,605). These results are consistent with our findings. While the design of the Berger et al. study was similar to the study design employed here, Berger et al. limited the index medication to sildenafil, whereas a variety of PAH-indicated medications were allowed as the index medication in the present study. Differences in cost comparisons among studies could also be due to inflation, as studies used different time periods for subject identification. Although we are not aware of studies from countries other than the US that have used a similar study design and could be directly compared to the results of this study, Wilkens et al. reported the overall treatment costs of PAH in Germany at 47,400 EUR per-patient-per-year (or about $65,000) among patients receiving treatment from 2004–2006, which is lower than total health care costs reported here [24].

Certain data limitations that are inherent to claims-based analyses should be considered when interpreting the results of this study. Claims data are collected for the purpose of payment, not research, and therefore capture an individual’s medical history to a limited degree. Claims data do not contain detailed information on symptoms, no self-reported burden of illness measures were obtained in this study, and quality of life measures were not available. Importantly, existing ICD-9-CM codes may not be specific enough to distinguish PAH from other related conditions, and it is therefore difficult to locate subjects with PAH in administrative claims. Based on exploratory analyses, we found that requiring evidence of a PAH-indicated medication appeared to be a more specific inclusion criterion than requiring PH/PAH-associated diagnosis codes. Average total health care costs among the PAH medication group and PAH diagnosis group were both lower than was observed among subjects in the final study cohort. This suggests that some of the subjects selected in these exploratory analyses may have had earlier/pre-treatment stages of PAH, and/or perhaps suggests that the selection criteria for these analyses were not specific enough, in comparison to the final algorithm. It is important to keep in mind that the PAH medication group and the PAH diagnosis group were performed as exploratory analyses; they each incorporated different subsets of selection criteria used for the final study cohort, but were not intended to be as specific as the final study cohort. For inclusion in the final study cohort, we used a claims-based algorithm requiring evidence of a PAH-indicated medication, PH diagnosis codes, and PAH-associated condition diagnosis codes, in order to increase specificity of selection. However, further research, such as a medical chart review, would be needed to validate the claims-based patient identification algorithm used here.

No comparisons were performed in this study using a control group that did not have PAH, and this is a further study limitation. As we required a period of 12 months of continuous enrollment following the index date for study inclusion, patients who died shortly following the index date would have been excluded from the study, and this may have impacted cost estimates. Also, measures from the 180 day baseline period were annualized, and this may have introduced some bias into the results. Cost and resource utilization estimates may also have been impacted by comorbid diseases. We observed high rates of some comorbid diseases in this population, such as systemic lupus erythematosus and respiratory infections, and this may have led to higher costs and utilization in some patients. Finally, the results observed in this study are most applicable to a managed care population diagnosed with and being treated for PAH, and may not be generalizable to other populations or to the US population in whole.

Additional limitations regarding pharmacy claims should be considered. For example, it is not possible to determine whether subjects consumed their medication as prescribed. In addition, over-the-counter medications and medications provided as samples by physicians cannot be located in claims data. A small proportion of study subjects (6%) had evidence of PAH medication use prior to the index date, and this may have affected comparisons of costs between the baseline and follow-up period (as on average lower total costs were observed after PAH medication use). We assumed that pharmacological treatment for PAH was a strong indication that physicians believed their patients to experience PAH (regardless of diagnosis coding), as the medications used are indicated only for the treatment of PAH (per the US package insert/ label, which note only PAH as an indication). However, this assumption is most at risk in the case of tadalafil and sildenafil, which are branded as Adcirca® and Revatio® for treatment of PAH only, but which are also formulations of generics that are branded as Cialis® and Viagra®, respectively, which are not indicated for PAH. Likewise, another caveat is that some off label use of Cialis® and Viagra® may occur, and patients who used these medications to manage PAH were not included in this study. Pharmacy claims for the managed Medicare population also present certain limitations. Prior to 2006, managed Medicare enrollees had pharmacy covered through their health plan, one of the benefits of enrollment in certain managed Medicare plans. However, for a portion of these enrollees, these pharmacy benefits had caps on expenditures. Enrollees with substantial pharmacy expenditures may have exceeded their cap in certain years. For these members, additional filled prescriptions may not be observed in the claims data, and it would appear that these individuals ceased to fill prescriptions. This limitation may have impacted the findings we report for treatment patterns.

## Conclusions

The results from this study indicate that medical costs decreased by nearly half from the baseline to the follow-up period (following evidence of use of PAH medication). However, annual total follow-up costs among PAH patients reported here were still substantively high on a per subject basis. Further, this study found that about half of subjects with PAH discontinued, switched, or augmented their index PAH medication. Further research is needed to examine whether the treatment patterns revealed in this study represent clinically optimal or cost-effective care.

## Additional file

Additional file 1: Table S1. Codes for PAH-Associated Conditions.
