# Supplementary material for: The economic burden of pulmonary arterial hypertension (PAH) in the US on payers and patients
Source: BMC Health Serv Res. 2014 Dec 24;14:676. doi: 10.1186/s12913-014-0676-0 (PMC4301626; doi:10.1186/s12913-014-0676-0)
Supplement: Additional file 1: Table S1. — Codes for PAH-Associated Conditions. [file 12913_2014_676_MOESM1_ESM.doc]

**Additional file 1: Table S1.** Codes for PAH-Associated Conditions

| **ICD-9-CM Diagnosis Codes** | **Description** |
| --- | --- |
| 710.x , 517.2, 714.0, 714.2, 714.3x, 714.4 | Connective tissue diseases |
| 745.0, 745.10, 745.3-745.5, 745.6x-745.7, 745.9, 747.0, 747.41-747.42, 745.0, 745.10, 745.11, 745.12, 745.19, 745.2, 745.3, 745.4, 745.5, 745.60, 745.61, 745.69, 745.7, 745.8, 745.9, 746.00, 746.01, 746.02, 746.09, 746.1, 746.2, 746.3, 746.4, 746.5, 746.6, 746.7, 746.81, 746.82, 746.83, 746.84, 746.85, 746.87, 746.89, 746.9 | Congenital heart diseases |
| 572.3 | Portal hypertension |
